# Conservation Implications for the Iberian Narrow Endemic *Androsace cantabrica* (Primulaceae) Using Population Genomics With Target Capture Sequence Data

**DOI:** 10.1002/ece3.71901

**Published:** 2025-08-08

**Authors:** Ke (Jungle) Liang, Amelia Shepherd‐Clowes, Agustí Agut, Oriane Hidalgo, Pablo Tejero Ibarra, Juan Viruel

**Affiliations:** ^1^ Royal Botanic Gardens, Kew Richmond UK; ^2^ Institute for Biodiversity and Ecosystem Dynamics (IBED) University of Amsterdam Amsterdam the Netherlands; ^3^ Banco de germoplasma del Jardín Botánico de Olarizu Vitoria‐Gasteiz Spain; ^4^ Institut Botànic de Barcelona (IBB) CSIC‐CMCNB Barcelona Spain; ^5^ Instituto Pirenaico de Ecología (IPE‐CSIC) Jaca Huesca Spain; ^6^ Aranzadi Zientzia Elkartea Donostia Spain

**Keywords:** alpine ecosystem, Angiosperms353, conservation genetics, global warming, phylogenomics, population genomics, threatened species

## Abstract

*Androsace cantabrica* (Losa & P. Monts.) Kress is a narrow endemic alpine plant restricted to a few high‐elevation localities in the Cantabrian Mountains of northern Spain. Although currently accepted as a distinct species, its close morphological and phylogenetic affinity to related taxa such as *Androsace adfinis* and *Androsace halleri* has led to historical uncertainty about its taxonomic status and evolutionary origin. Here, we use the universal Angiosperms353 target capture kit to generate nuclear and plastid data from *A. cantabrica* and closely related species in section *Aretia*. We employ phylogenomic analyses to clarify species boundaries and population genomic analyses to inform conservation management, as well as flow cytometry and sequence‐based analysis using allelic frequencies to estimate its ploidy level. Phylogenetic analyses based on nuclear loci support *A. cantabrica* as a monophyletic clade, distinct from both *A. adfinis* and the *A. halleri* and *Androsace laggeri* clades, although topological incongruence with plastid data suggests historical hybridization. Flow cytometry and allelic frequency‐based analysis indicate that *A. cantabrica* is tetraploid, differentiating it from *A. halleri* and *A. laggeri*, which are diploid. Population structure analyses reveal a shallow genetic split between eastern and western groups (*F*_ST_ = 0.04485), with higher genetic diversity observed in the east. We estimated the species' distribution, population sizes, and threats, and classified it as Vulnerable under IUCN criteria B1ab(ii,iii) + 2ab(ii,iii). We recommend targeted in situ management, *ex situ* seed conservation, and the establishment of a micro‐reserve. This study illustrates the utility of Angiosperms353 data for resolving both taxonomic questions and conservation strategies in polyploid, range‐restricted species.

## Graphical abstract

This study uses Angiosperms353 target capture sequencing to resolve the taxonomic status and conservation needs of *Androsace cantabrica*, a rare alpine plant endemic to northern Spain. Phylogenomic analyses confirm its distinctiveness from related species, and population genomic data reveal shallow genetic structure and moderate diversity, supporting its classification as Vulnerable under IUCN criteria. The findings advocate for targeted in situ and *ex situ* conservation actions, including habitat management and seed banking.

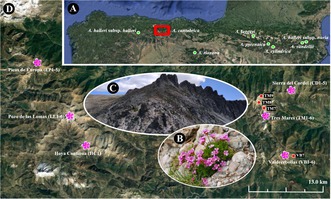

## 1 Introduction

Alpine environments, dominated by perennial herbs, face severe impacts from global climate change (Seddon et al. 2016). Global warming has led to significant changes, such as the encroachment of woody subalpine plants, narrowing of alpine ecosystems (Capers and Stone 2011), or an increase of diversity in European summits (Steinbauer et al. 2018). While the loss of alpine habitat could be compensated by glacier retreat (Whittaker 1993; Losapio et al. 2021), the snowline has been completely lost in the lower and southern mountain regions, where alpine plants now persist only on ridges and peaks, facing potential local extinction (Rumpf et al. 2022). Global warming also affects alpine plant reproduction, including plant–pollinator interactions (Inouye 2020) and seed germination (Mondoni et al. 2012). Moreover, human disturbance in mountainous areas, such as civil infrastructure or recreational development, causing fragmentation and degradation of alpine habitats (Winkler 2020; Chardon et al. 2023). Therefore, alpine ecosystems and their species are considered especially vulnerable to global warming, which is driving many of these documented changes (Schwager and Berg 2019). However, some authors suggest that due to the high microhabitat heterogeneity characteristic of alpine environments, many mountain plant species may exhibit a degree of resilience to warming through short‐distance range shifts (Tanneberger et al. 2021; Körner and Hiltbrunner 2021).

By 2100, 36%–55% of the alpine species in European mountains are predicted to lose more than 80% of their habitats (Inouye 2020). However, limited information is available regarding the current conservation status of many European alpine species, such as those included in the *Androsace* L. section *Aretia* (L.) W.D.J. Koch. Section *Aretia* includes narrow endemics with low dispersal ability (Anderberg and Kelso 1996), with 34 recognized species (Boucher et al. 2021), mainly distributed in the “European Alpine System” (Ozenda 1995). Only a handful of *Aretia* species have undergone threat assessments (Fasciani and Pace 2015; Eustacchio et al. 2023). In Spain, *Androsace cantabrica* (Losa & P. Monts.) Kress has been included in the list of priority species for conservation (Moreno Saiz et al. 2008).

**FIGURE 1:**
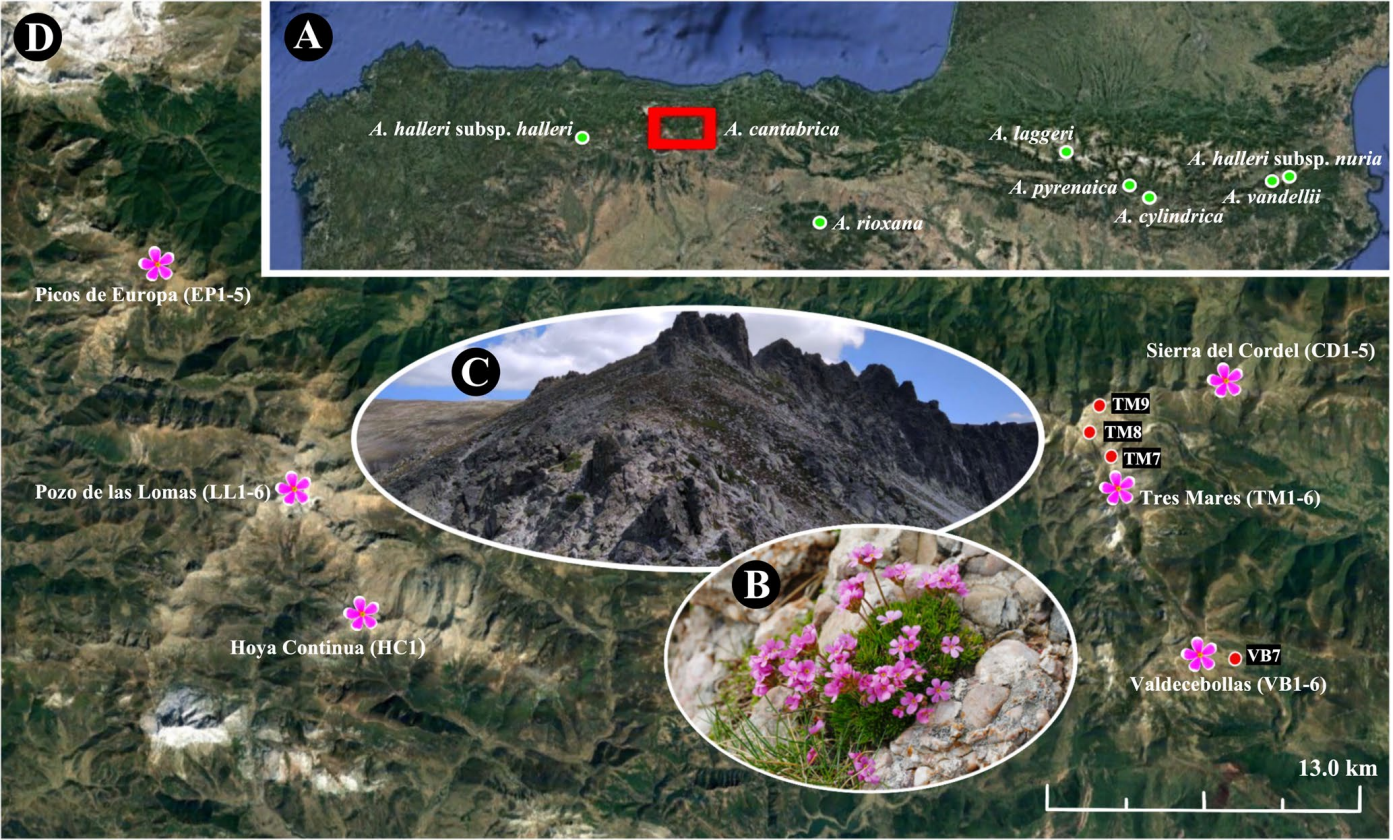
(A) Distribution and sampling sites of *A. cantabrica* and related taxa in the northern Iberian Peninsula. (B) Field photo of *A. cantabrica*. (C) Natural habitat of *A. cantabrica*. (D) Six known distribution sites of *A. cantabrica* with information and coding on population and individual sampling.

*Androsace cantabrica* is an alpine plant endemic to the Cantabrian Mountains of northern Spain (Figure 1A), initially described as a distinct species based on morphological and cytological evidence (Kress 1997, 2022). It is a perennial, monoecious plant with small, densely clustered rosettes. The peduncle is usually less than 5 cm long, and the flower corolla is deep pink (Figure 1B; Kress 1997). This species is found on siliceous or acidic substrates in mountainous areas above 2,000 m, typically in ridges, and often associated with low shrubs and alpine pastures (Figure 1C; Tejero et al. 2022). *A. cantabrica* is known to occur in six localities (Figure 1D), with less than 6000 individuals estimated across twenty 1 × 1 km UTM quadrats reported by Baudet et al. (2004). The distribution is centered on the Tres Mares area, partially overlapping with a ski resort. All population sites have traditionally been subjected to controlled burning to promote pasture development. Global warming is likely to affect its reproductive output, as Tejero et al. (2022) observed lower germination rates in experiments conducted at warmer temperatures. Baudet et al. (2004) proposed categorizing *A. cantabrica* as “Endangered” in the Spanish Red List, which was later confirmed by Moreno Saiz et al. (2008).

Despite its clear geographical and ecological separation from related taxa, the taxonomic status of *A. cantabrica* has remained uncertain due to occasional morphological overlap with *A. halleri* and potential hybridization events. Recent morphological reassessments (Kress 2022) reaffirm the distinctiveness of the *A. cantabrica* morphotype while also raising the possibility that *A. cantabrica* and *A. halleri* may indeed co‐occur in the Peña Prieta region, which was previously considered unlikely. Ploidy levels remain another unresolved aspect; earlier studies by Kress estimated *A. cantabrica* as octoploid (2*n* ≈ 76), contrasting with a tetraploid *A. halleri* (2*n* = 38). However, conflicting chromosome counts in the literature may stem from misidentification of specimens (Kress 2022). In addition, earlier morphological comparisons suggested a close relationship between *A. cantabrica* , *A. halleri* , and *A. laggeri* A. Huet (Kress 1997). Genetic studies using various molecular markers, including the plastid *trn*L‐F region and the internal transcribed spacer (ITS, Schneeweiss et al. 2004), amplified fragment length polymorphism (AFLPs, Dixon et al. 2008), and double digest restriction‐site associated DNA (ddRADseq, Boucher et al. 2021), have proposed that *A. cantabrica* and *A. adfinis* Biroli *s.l*. are sister taxa. Together, they form a *cantabrica‐adfinis* clade, which is sister to a clade comprising *A. halleri* and *A. laggeri* (hereafter referred to as the /halleri clade), although these relationships were recovered with low bootstrap support. More recently, phylogenetic analyses based on complete plastome sequences have suggested that the *cantabrica‐adfinis* clade may not be sister to the /halleri clade (Smyčka et al. 2022). Given these uncertainties, robust scientific evidence is urgently needed to delimit *A. cantabrica* as a distinct conservation unit, an essential first step toward developing effective conservation strategies (Godfray et al. 2004). Phylogenomic approaches that integrate nuclear and plastome sequence data provide a powerful framework to resolve their taxonomic status and evolutionary relationships, particularly in the presence of conflicting phylogenetic signals among different molecular markers.

Targeted sequencing using the universal Angiosperms353 probe set can generate hundreds of homologous low‐copy nuclear sequences, establishing it as a powerful tool in plant evolutionary studies (Johnson et al. 2019). This approach is cost‐effective and allows the use of herbarium materials in phylogenomic analysis (Brewer et al. 2019). Nuclear genes can yield a distinct phylogenetic topology compared to plastid genes (Stubbs et al. 2023). By combining these two genomic sources, researchers can explore reticulate evolution and potential hybrid origins more effectively (Vriesendorp and Bakker 2005). However, most previous studies using Angiosperms353 data have primarily focused on clade boundaries at the genus, family, and order levels (e.g., *Nepenthes* (Nepenthaceae), Murphy et al. 2020; Gentianales, Antonelli et al. 2021; Primulaceae, Larson et al. 2023), with few addressing species‐level taxonomic conflicts (e.g., Campos et al. 2023). In addition to its application in phylogenomics, Angiosperms353 data can be utilized in population genetic studies (Slimp et al. 2021), which is invaluable for designing effective conservation plans for threatened species (Liu and Zhao 1999; Xiong et al. 2024). Compared to RADseq (Davey and Blaxter 2010), Angiosperms353 offers a more cost‐effective alternative with reduced missing data, and it can be used in plants without requiring an optimization of the protocol for different genome sizes (Slimp et al. 2021). However, to our knowledge, the application of Angiosperms353 in practical conservation genetics has yet to be reported.

Our research objectives are threefold: to assess whether *A. cantabrica* forms a monophyletic clade, distinct from *A. halleri* and other close relatives, using phylogenomic data; to evaluate its threatened status and IUCN category; and to provide conservation recommendations for *A. cantabrica* based on population genetic analyses. This approach will also enable us to evaluate the effectiveness of Angiosperms353 in conservation genetics research.

## 2 Materials and Methods

### 2.1 Plant Material

In the summer of 2020, *A. cantabrica* populations were sampled from six locations that collectively represent its distribution range, as illustrated in Figure 1D. Within these populations, its distribution is often fragmented, resulting in multiple subpopulations, notably in Tres Mares (TM). A total of thirty‐five individuals were collected, with six individuals sampled from each population (individual codes within each population are shown in Figure 1D, with one individual from Picos de Europa (EP) and one from Sierra del Cordel (CD) discarded due to low gene recovery), except for Hoya Continua (HC), where only one individual was available for analysis. Three additional individuals were sampled from different subpopulations within TM (TM7‐9), along with one more from a separate Valdecebollas (VB) subpopulation (VB7). Related taxa of *A. cantabrica* from the northern Iberian Peninsula were also sampled, including six individuals from an *A. halleri* subsp. *nuria* Schönsw. & Schneew. population, one sample of *A. halleri* subsp. *halleri*, one sample of *A. laggeri*, one sample of *A. vandellii* Chiov., one sample of *A. pyrenaica* Lam., one sample of *A. cylindrica* subsp. *hirtella* (L.Dufour) Greuter & Burdet, and one sample of *A. rioxana* A.Segura (Figure 1A; SRA ERR7620530). Fresh leaf tissue samples were dried in silica gel and preserved in the JACA Herbarium together with the corresponding voucher (Table 1). Specimen material from the four alpine taxa was obtained from the Kew Herbarium (*Androsace alpina* (L.) Lam., K006548304; *A. adfinis* subsp. *adfinis*, K006547847; *A. adfinis* subsp. *brigantiaca* (Jord. & Fourr.) Kress, K001684072; *A. adfinis* subsp. *puberula* (Jord. & Fourr.) Kress, K006547862). Additional sequence data were downloaded from public repositories for *Androsace spinulifera* (Franch.) R.Knuth (SRR19354411), *A. sarmentosa* subsp. *primuloides* (Hook.f.) Govaerts (ERR7620526), *A. vitaliana* (L.) Lapeyr. (ERR7620605), and *Primula matthioli* (L.) V.A.Richt, which was selected as an outgroup. During the field survey and sampling, we estimated the population size of the visited populations by the total count method to complement previous studies (Baudet et al. 2004). We further reassessed the IUCN category following the standards set by the IUCN Species Survival Commission (2012).

**TABLE 1:** Field sampling information for *Androsace cantabrica* and related taxa samples.

Species	*N*	Sequence ID	Herbarium	Coordinates	Altitude (m)	Location	Date	Collector(s)
*A. cantabrica*	6	21B51‐21B56	JACA‐R308670	−4.352874, 42.968251	2044	Valdecebollas (VB), Brañosera	28.07.2020	Pablo Tejero
*A. cantabrica*	6	21B57‐21B62	JACA‐R308667	−4.799987, 43.088023	2084	Picos de Europa (EP), Camaleño	31.07.2020	Pablo Tejero
*A. cantabrica*	6	21B63‐21B68	JACA‐R308672	−4.387157, 43.019988	2087	Tres Mares (TM), Campo de Suso	07.07.2020	Pablo Tejero
*A. cantabrica*	6	21B69, 21B86‐21B89	JACA‐R308668	−4.748242, 43.016833	2331	Las Lomas (LL), Velilla del río Carrión	29.07.2020	Pablo Tejero
*A. cantabrica*	6	Z1‐Z2, Z4‐Z7	JACA‐R309275	−4.344132, 43.049344	2036	Sierra del Cordel (CD), Campo de Suso	15.08.2021	Pablo Tejero
*A. cantabrica*	1	Z8	JACA‐R309277	−4.714069, 42.984311	2172	Hoya Continua (HC), Velilla del río Carrión	15.08.2021	Víctor Ezquerra & José Vicente Ferrández
*A. cantabrica*	1	21B47	JACA‐R308692	−4.338801, 42.971271	1948	Peña Astia (VB), Brañosera	28.07.2020	Pablo Tejero
*A. cantabrica*	1	21B48	JACA‐R308671	−4.392118, 43.027195	2147	Tres Mares (TM), Campo de Suso	07.07.2020	Pablo Tejero
*A. cantabrica*	1	21B49	JACA‐R308694	−4.401904, 43.040843	2047	Tres Mares (TM), Campo de Suso	07.07.2020	Pablo Tejero
*A. cantabrica*	1	21B50	JACA‐R308693	−4.398327, 43.034692	2051	Tres Mares (TM), Campo de Suso	07.07.2020	Pablo Tejero
*A. halleri* subsp. *nuria* Schönsw. & Schneew.	6	21B79‐21B84	JACA‐R308666	2.185143, 42.396624	2739	Queralbs	09.08.2020	Pablo Tejero, Víctor Ezquerra & José Vicente Ferrández
*A. halleri* subsp. *halleri* L.	1	21B85	JACA‐R308661	−5.494311, 43.017505	2129	Cármenes	30.09.2020	Pablo Tejero, Víctor Ezquerra & José Vicente Ferrández
*A. laggeri* A.Huet	1	21B70	JACA‐R308680	−0.437182, 42.765285	2525	Canfranc	24.05.2020	Víctor Ezquerra
*A. vandellii* Chiov.	1	21B44	JACA‐R308674	2.136649, 42.405635	2270	Queralbs	10.08.2020	Pablo Tejero, Víctor Ezquerra & José Vicente Ferrández
*A. pyrenaica* Lam.	1	21B45	JACA‐R308660	0.071591, 42.572477	2000	Puertolas	06.08.2020	Pablo Tejero
*A. cylindrica* subsp. *hirtella* (L.Dufour) Greuter & Burdet	1	21B46	JACA‐R308673	0.200231, 42.493552	1900	Laspuña	06.08.2020	Pablo Tejero
*A. rioxana* A.Segura	1	21E24	JACA‐R309142	−2.971394, 42.244646	2149	Circo San Lorenzo	12.06.2021	Pablo Tejero & Joseba Garmendia

*Note: N*, number of samples.

### 2.2 Molecular Methods to Generate Angiosperms353 Sequence Data

Total DNA was isolated using a modified CTAB protocol (Doyle and Doyle 1987). Genomic libraries were constructed as optimized in Viruel et al. (2019) using half volumes of the NEBNext UltraTM II DNA Library Prep Kit for Illumina (New England Biolabs, Ipswich, MA, United States), purified using AMPure XP magnetic beads, and multiplexed with NEBNext Multiplex Oligos for Illumina (Dual Index Primer Sets I and II). Equimolar pools containing 12 genomic libraries were enriched with half‐reactions of the Angiosperms353 probe kit (Johnson et al. 2019; Baker et al. 2022) following the myBaits kit manual v5.03 (Arbor Biosciences). DNA concentrations were determined using a QuantusTM fluorometer (Promega Corp.). Fragment length was assessed using an Agilent 4200 TapeStation (Agilent Technologies, Santa Clara, CA, United States). Sequencing was performed on a HiSeq (Illumina Inc.) by Macrogen (Seoul, South Korea), producing 150 bp paired‐end reads. Sequence data from this study are available at the ENA repository PRJEB90944.

### 2.3 Quality Filtering of FASTQ Raw Data

The raw sequencing files were checked for quality using FastQC (Andrews 2010) and MultiQC (Ewels et al. 2016), then trimmed using Trimmomatic (Bolger et al. 2014) to remove adapters and reads with low quality (LEADING:30 TRAILING:30). Paired reads were used as input in HybPiper (Johnson et al. 2016), and the mega353 target file (McLay et al. 2021) was used to recover Angiosperms353 loci sequences. Reads were mapped to the mega353 reference using BWA (Li and Durbin 2009) and then assembled *de novo* using SPAdes (Bankevich et al. 2012). Exon, intron, and supercontig sequences were recovered using Exonerate (Slater and Birney 2005). We excluded genes flagged with paralog warnings by HybPiper and genes that were not recovered in at least 75% of samples.

We extracted protein‐coding and intergenic sequences from the complete plastid genome of *Androsace mariae* Kanitz (GenBank: MT732944) and removed duplicates and sequences shorter than 200 bp, resulting in a plastome reference of 125 plastid fragments. This reference was then used to recover plastid sequences with HybPiper, as described above.

### 2.4 Estimation of Ploidy

Nuclear DNA content was estimated for three individuals of *A. cantabrica* and one of *A. laggeri* using a CyFlow Space flow cytometer (Sysmex‐Partec, Norderstedt, Germany) equipped with a 100‐mW green solid‐state laser (Cobolt Samba). The procedure followed the one‐step protocol of Doležel et al. (2007), with modifications as described by Clark et al. (2016). Samples were prepared with the “general purpose buffer” (GPB; Loureiro et al. 2007) supplemented with 3% polyvinylpyrrolidone (PVP‐40). Fresh leaf tissue from *Androsace* individuals was used, with *Solanum lycopersicum* L. “Stupické polní rané” (1.96 pg/2C; Doležel et al. 1992) serving as an internal standard for *A. laggeri*, and *Petroselinum crispum* “Champion Moss Curled” (4.5 pg/2C; Obermayer et al. 2002) for *A. cantabrica* . Each individual was measured twice. For each run, nuclear DNA content was estimated by analyzing a minimum of 1000 nuclei per fluorescence peak. The resulting histograms were processed using FlowMax software (v. 2.9, Sysmex‐Partec GmbH).

We also estimated the ploidy levels of *Androsace* samples using nQuire (Weiß et al. 2018) following the approach by Viruel et al. (2019). To prepare the reference file for nQuire, we extracted the longest exon recovered per gene using bioawk (available at https://github.com/lh3/bioawk) and excluded any genes that received paralog warnings from the initial reference. We then evaluated ploidy by analyzing the delta log‐likelihood (*Δ*log*L* values produced by nQuire across three models—diploid, triploid, and tetraploid—to identify the best‐supported ploidy level).

### 2.5 Phylogenomic Analysis

We reconstructed phylogenetic trees using one representative sample from each of the six *A. cantabrica* populations and all other taxa. Genome skimming data available in public repositories for *Androsace spinulifera* (SRR19401086), *A. adfinis* subsp. *adfinis* Biroli (ERR9124249), *A. adfinis* subsp. *brigantiaca* (Jord. & Fourr.) Kress (ERR9124250), *A. adfinis* subsp. *puberula* (Jord. & Fourr.) Kress (ERR9124251) and *A. alpina* (L.) Lam. (ERR9124252) were incorporated in the analysis to reconstruct plastid phylogenetic trees.

Loci sequences were aligned with MAFFT (‐‐auto; Katoh and Standley 2013), and then the alignments were trimmed with trimAl (‐automated1; Capella‐Gutiérrez et al. 2009). For both the Angiosperms353 loci and the 125 recovered plastid fragments, we inferred phylogenetic trees using both coalescent and concatenated maximum likelihood (ML) approaches. In the coalescent approach, we inferred single‐locus phylogenetic trees from the corresponding trimmed alignments using IQ‐TREE (Minh et al. 2020) with 1000 ultrafast bootstrap replicates (‐bb 1000; Hoang et al. 2018), and branches with less than 10% bootstrap support were collapsed with Newick utilities (Junier and Zdobnov 2010). We then used ASTRAL‐III (Zhang et al. 2018) to infer the species tree (hereafter, ASTRAL tree), applying the “‐t 3” flag to annotate local posterior probabilities (LPP) for each node.

In the concatenated ML approach, all trimmed alignments were concatenated with FASconCAT‐G (Kück and Meusemann 2010). The best‐fit model inferred by IQ‐TREE (‐m MFP) was applied in RAxML‐NG (Kozlov et al. 2019) to infer the species tree using the concatenated partitioned matrix with 1000 bootstrap replicates (‐‐tree pars{20} ‐‐bs‐trees 1000; hereafter, RAxML tree). Additionally, we implemented a greedy strategy (Lanfear et al. 2012) with the relaxed hierarchical clustering algorithm (Lanfear et al. 2014) to select the best partition model, which was applied in IQ‐TREE to infer the species tree with 1000 SH‐like approximate likelihood ratio test replicates (‐alrt 1000; Guindon et al. 2010) and 1000 ultrafast bootstrap replicates (hereafter, IQ‐partition tree). We visualized the phylogenetic trees using Dendroscope (Huson and Scornavacca 2012) and FigTree (available at https://github.com/rambaut/figtree).

To investigate potential phylogenetic conflicts and signs of reticulate evolution, we used SplitsTree4 (Huson and Bryant 2006) to create a split network based on the Neighbor‐Joining algorithm with the Angiosperms353 data. In the resulting network, we masked specific samples to retain only those within the *A. cantabrica* , *A. adfinis* , and *A. halleri* clades for focused analysis.

### 2.6 Variant Calling and Filtering

To compare the population genetic results between the threatened *A. cantabrica* and the nonthreatened *A. halleri* subsp. *nuria*, we performed variant calling and population genetic analyses using 33 *A. cantabrica* samples from six populations and six samples of *A. halleri* subsp. *nuria* from a single population. We followed the pipelines and scripts provided by Slimp et al. (2021, available at https://github.com/lindsawi/HybSeq‐SNP‐Extraction) and the framework developed by DePristo et al. (2011) in GATK (McKenna et al. 2010), with some modifications. In their pipeline, Slimp et al. (2021) used supercontig sequences, demonstrating that most genetic variation occurred in flanking noncoding regions, which tend to accumulate mutations quickly due to limited functional constraints (Palumbi 1996). To obtain more accurate and representative single‐nucleotide polymorphism (SNP) data, we restricted SNP calling to noncoding regions for downstream analyses. We called SNPs for *A. cantabrica* and *A. halleri* separately, using the longest intron sequence of each gene for all individuals of each species as a reference. We combined aligned and unaligned reads to the reference, removed duplicate sequences, and performed genotype calling collectively for all samples after generating preliminary variants individually for each sample (Poplin et al. 2018) in a Variant Call Format (VCF) file. We set the parameter “‐ploidy 4” for tetraploid *A. cantabrica* in “gatk HaplotypeCaller” Additionally, we conducted a Base Quality Score Recalibration in GATK and repeated the variant calling step. The filtering conditions we conducted on the initial VCF file included using a “hard filter” (QD < 2.0, QUAL < 50.0, GQ < 5.0, FS > 60.0, SOR > 3.0, MQ < 40.0, MQRankSum < −12.5, ReadPosRankSum < −8.0), removing indels, retaining 20% missing data, and performing pruning of linkage disequilibrium (LD; ‐m r2 = 0.2, ‐w 50 kb in BCFtools). To address the potential effects of polyploidy, which can artificially increase heterozygosity and allelic richness (Hokanson and Hancock 1998), it is essential to filter fixed heterozygotes in SNP datasets in polyploid species (e.g., Douglas et al. 2015; Cornille et al. 2016; Blischak et al. 2018). We used the HDPlot method (H: frequency of heterozygotes; D: deviation from the expected proportion of reads; McKinney et al. 2017) to remove SNPs with unusually high heterozygosity and extreme allelic imbalance, with filtering parameters of *H* > 0.7, abs(D) > 15.

### 2.7 Population Genetic Indicators for Conservation Recommendations

Following the framework proposed by Ottewell et al. (2016) for conservation planning, we calculated three population genetic indicators: genetic differentiation (*F*_ST_), genetic diversity (observed and expected heterozygosity, *H*_O_ and *H*_E_), and inbreeding coefficient (*F*_IS_) using the R packages *adegenet* (Jombart 2008), *APE* (Paradis et al. 2004), and *hierfstat* (Goudet 2005). To identify conservation management units and set conservation priorities, we analyzed the population genetic structure of *A. cantabrica* as outlined by Fraser and Bernatchez (2001) using three primary approaches: (1) Principal Coordinate Analysis (PCoA): We generated genetic distance‐based PCoA plots using the R package *ggrepel* (Slowikowski et al. 2024) to visualize genetic relationships among populations; (2) Split Network Analysis: We converted the filtered VCF file into a distance matrix file in VCF2Dis (available at https://github.com/BGI‐shenzhen/VCF2Dis) and then applied it to SplitsTree using Neighbor‐Net; (3) Clustering Analysis: We inferred the optimal number of genetic clusters (*K*) using STRUCTURE (Pritchard et al. 2000) using 100,000 burn‐in and 1,000,000 MCMC generations, with 10 replicates per *K* value, testing up to *K* equal to the number of populations plus two. The most likely *K* was determined following the Evanno et al. (2005) approach as implemented in Structure Harvester (Earl and von Holdt 2012), and the results were visualized with StructuRly (Criscuolo and Angelini 2020).

### 2.8 Germination Conditions for *Androsace cantabrica*

The following six germination protocols, involving combinations of cold stratification and alternating temperature treatments, were tested using seeds of *A. cantabrica* collected in Tres Mares (TM): stratification (Yes/No) × temperature night/day simulation cycles (4°C–14°C, 12°C–22°C, and 20°C–30°C). For each treatment, five Petri dishes were set with 10 seeds in each. In total, 300 seeds were cultivated, with 50 seeds in each of the treatment combinations. To stimulate germination, seeds were incubated for 1 day in a 250‐ppm solution of gibberellic acid (GA3, 90% purity) before cultivation in Petri dishes containing 1% agar.

## 3 Results

### 3.1 Phylogenetic Trees and Network

On average, 32.98% of reads were mapped to target regions, ranging from 14.88% to 66.83%. The sequence length recovery rate, relative to gene lengths in the mega353 target file, averaged 80.56%, ranging from 25.55% to 86.52% (Table S1). One individual in the EP population and one sample in CD were removed from the population analysis due to their low gene recovery. The average plastid sequence length recovery rate was 92.03%, ranging from 60.69% to 98.64%, excluding herbarium samples (Table S2).

**FIGURE 2:**
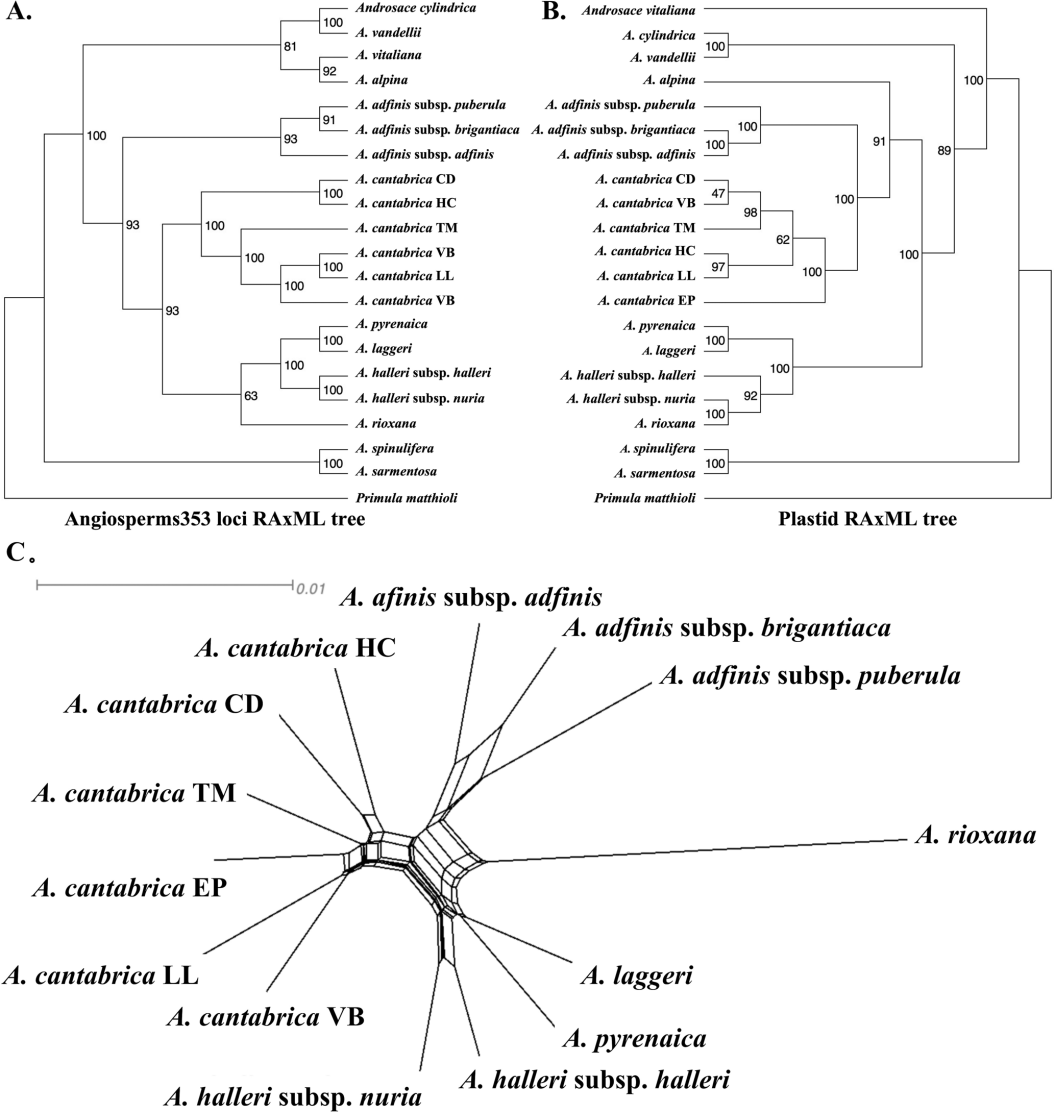
Phylogenetic tree and network split plots. (A) Angiosperms353 loci RAxML tree with node values indicating support from 1000 bootstrap replicates. (B) RAxML tree of 125 plastid fragments derived from Angiosperms353 off‐target data. *A. adfinis* and *A. alpina* plastid sequences are derived from the online genome skimming data. (C) Angiosperms353 loci phylogenetic network split plot.

The phylogenetic trees reconstructed with the Angiosperms353 loci (hereafter, Angiosperms353 trees) show strong support, with bootstrap values above 90% for most interspecies nodes (Figure 2A and Figure S1). The IQ‐partition and RAxML trees are largely congruent, with *A. cantabrica* resolved as a sister to the /halleri clade (Figure 2A). In the ASTRAL tree, a phylogenetic conflict was observed regarding the placement of *A. rioxana* (Figure S1); here, *A. rioxana* was resolved as a sister to the /halleri and *A. cantabrica* clades rather than being embedded within the /halleri clade, as seen in other phylogenetic trees but with low support (Figure 2A). In the plastid tree, *A. cantabrica* is resolved as a sister to *A. adfinis* , with these two forming a clade that is sister to *A. alpina* . The /halleri clade is resolved as a sister to the clade formed by *A. alpina* , *A. adfinis* , and *A. cantabrica* (Figure 2B and Figure S2). *A. cantabrica* emerges as a relatively independent evolutionary branch related to *A. adfinis* and the /halleri clade in the SplitsTree analysis (Figure 2C).

### 3.2 Genome Size and Ploidy Level Estimation

The genome size of *A. laggeri* was estimated by flow cytometry at 1.23 pg/2C (standard deviation SD = 0.01; coefficient of variation of the plant (CV_plt_) = 3.97%; coefficient of variation of the standard (CV_std_) = 2.75%), while that of *A. cantabrica* was 2.39 pg/2C (SD = 0.01; CV_plt_ = 3.83%; CV_std_ = 2.81%).

In the sequence‐based ploidy estimation analyses, *A. cantabrica* samples consistently had the lowest Δlog*L* under the tetraploid model in nQuire (Table S3), and the same result was obtained for *A. rioxana* and *A. adfinis* subsp. *brigantiaca*. By contrast, the lowest Δlog*L* corresponding to a diploid model in nQuire was estimated for *A. halleri* subsp. *halleri*, *A. halleri* subsp. *nuria*, *A. laggeri*, *A. adfinis* subsp. *adfinis*, *A. adfinis* subsp. *puberula*, and *A. pyrenaica* (Table S3).

### 3.3 Population Genetic Analysis

**FIGURE 3:**
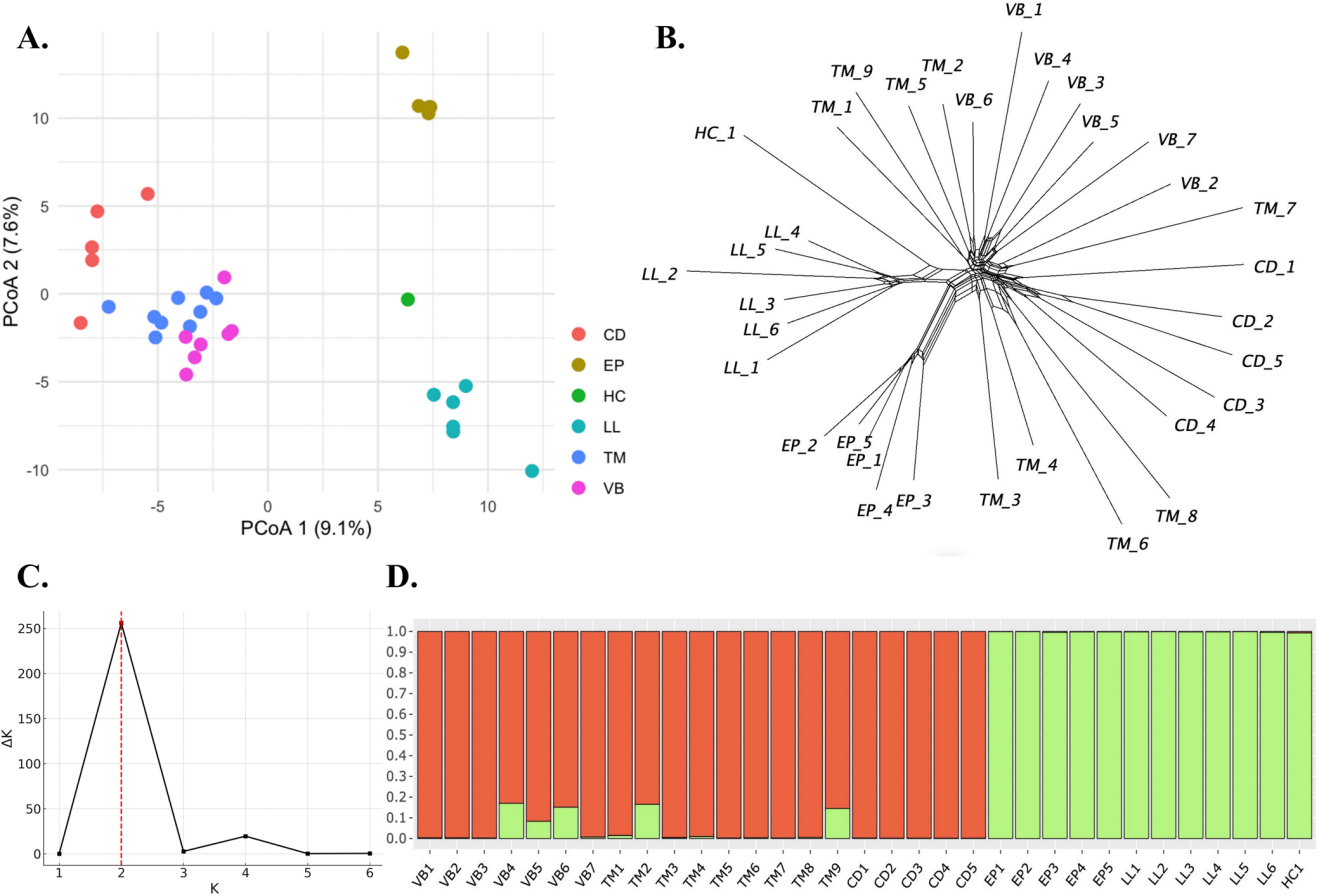
Results of population structure analyses within *A. cantabrica*. (A) PCoA plots. (B) Network split. (C) Delta*K* plot, generated using Structure Harvester from the STRUCTURE outputs, with the corresponding *K* values at their peaks on the plots representing the optimal cluster status for population structure (Evanno et al. 2005). (D) Population structure plots of all individuals are generated in StructuRly.

The VCF files initially contained 8987 variants for *A. cantabrica* and 2563 for *A. halleri* (SNPs and indels), respectively. After removing indels and applying a hard filter, 7947 and 2339 SNPs remained. Subsequent filtering using the HDPlot method reduced the datasets to 2494 and 657 SNPs. After allowing up to 20% missing data and performing linkage disequilibrium pruning, the final datasets comprised 663 and 209 SNPs, respectively. Genetic structure analyses of *A. cantabrica* (Figure 3) revealed a clear separation between western populations (LL, EP, HC; Group W) and eastern populations (TM, VB, CD; Group E), supported by PCoA, split network, and clustering analyses. Pairwise *F*_ST_ values among populations ranged from 0.0153 to 0.1110 (Table 2), with the lowest values observed within Group E and the highest between the western and eastern populations. The overall differentiation between Group W and Group E was moderate (*F*_ST_ = 0.0448; Table 2).

**TABLE 2:** Genetic differentiation (*F*_ST_) values between the five location populations and the two groups within *A. cantabrica*.

Populations	TM	VB	CD	LL	EP	Group W	Group E
TM	—					—	—
VB	0.0153	—				—	—
CD	0.0351	0.0571	—			—	—
LL	0.0680	0.0722	0.1071	—		—	—
EP	0.0820	0.0988	0.1110	0.1019	—	—	—
Group W	—	—	—	—	—	—	—
Group E	—	—	—	—	—	0.0448	—

Abbreviations: CD, Sierra del Cordel; EP, Picos de Europa; LL, Pozo de las Lomas; TM, Tres Mares; VB, Valdecebollas.

Genetic diversity measures (Table 3) showed that *A. cantabrica* populations had lower observed (*H*_O_) and expected heterozygosity (*H*_E_) than the nonthreatened diploid *A. halleri* (*H*_O_ = 0.2910; *H*_E_ = 0.3564). Within *A. cantabrica*, Group E populations exhibited slightly higher *H*_E_ values (mean *H*_E_ = 0.2435) than Group W (mean *H*_E_ = 0.2348), with the TM population showing the highest *H*_E_. All *A. cantabrica* populations showed negative inbreeding coefficients (*F*_IS_), indicating excess of heterozygosity and low inbreeding risk (mean *F*_IS_ = −0.0632). In contrast, *A. halleri* had a positive *F*_IS_ (0.1553), suggesting a potential deficit of heterozygotes.

**TABLE 3:** Sample size (*N*), observed heterozygosity (*H*_O_), expected heterozygosity (*H*_E_) and inbreeding coefficients (*F*_IS_) values in *A. cantabrica* and *A. halleri* studied populations and groups.

Populations	*N*	*H*_O_	*H*_E_	*F*_IS_
*A. halleri*	6	0.2910	0.3564	0.1553
*A. cantabrica*				
TM	6	0.2641	0.2428	−0.0529
VB	6	0.2664	0.2282	−0.1175
CD	5	0.2619	0.2361	−0.0987
LL	6	0.2831	0.2254	−0.2063
EP	5	0.2753	0.2147	−0.2477
Mean	28	0.2702	0.2294	−0.1446
Group E	21	0.2668	0.2435	−0.0105
Group W	12	0.2772	0.2348	−0.1160
Mean	33	0.2720	0.2392	−0.0632

### 3.4 Germination of *A. cantabrica*

**FIGURE 4:**
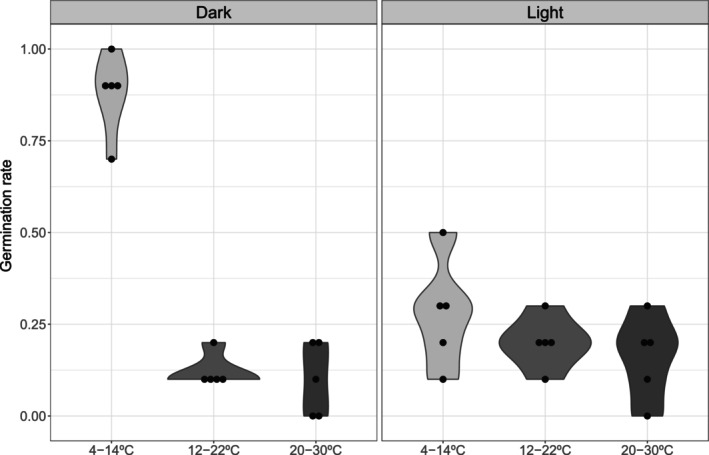
Germination responses of *A. cantabrica* seeds under different stratification treatments. Violin plots show the distribution of germination rates across replicates for each treatment, with treatments grouped by light regime (dark vs. light) along the *x*‐axis. The vertical axis indicates the final germination rate. Each violin represents the probability density of the data, with wider sections indicating a higher frequency of germination values. Dark and light treatments were applied under controlled temperature and moisture conditions to simulate alpine germination cues.

Our germination experiment shows that gibberellins significantly enhance the in vitro germination of *A. cantabrica* seeds. However, notable germination only occurred under the coldest treatment conditions, regardless of whether gibberellin was applied. The results indicate that *A. cantabrica* germination is strongly dependent on cold temperatures and darkness, with only marginal germination observed at higher temperatures and in the absence of dark stratification (Figure 4).

### 3.5 Conservation Status

As detailed in Table 4, our field observations indicate that current population sizes are lower than those previously informally observed. In the TM area in particular, the number of individuals encountered during surveys was noticeably low; while no systematic historical population data are available for direct comparison, this apparent reduction may be linked to increased anthropogenic pressures such as ski resort development and intensified recreational use of hiking trails. The current total number of individuals in all known ranges is estimated to be fewer than 6000 individuals.

**TABLE 4:** Estimated population sizes and threat factors of *A. cantabrica* populations.

Location	Number of individuals ever reported	Number of observed individuals	Threat factors
TM	3216	ca. 2500	Global warming^a^. Anthropogenic disturbances^b^
VB	109	ca. 100	Population size. Global warming
CD	417	ca. 300	Global warming
LL	1629^c^	ca. 100	Population size. Global warming
HC	—	ca. 1000	Global warming
EP	—	ca. 25	Population size. Global warming

*Note:* LL is a subpopulation of Peña Prieta.

^a^ Shrubs and heath are colonizing mountain peaks and ridges.

^b^ Ski resorts and hiking trails.

^c^ This number corresponds to the entire Peña Prieta population.

Based on our distribution range and population size results, we propose that *A. cantabrica* be classified as Vulnerable (VU) under the IUCN Red List criteria: B1ab(ii,iii) + 2ab(ii,iii). This categorization is supported by an estimated extent of occurrence (EOO) of less than 20,000 km^2^ (B1); an area of occupancy (AOO) of less than 2000 km^2^ (B2); fewer than 10 locations (a), and ongoing declines in both AOO (b(ii)) and habitat extent and quality (b(iii)).

## 4 Discussion

### 4.1 Species Boundary Delimitation of *Androsace cantabrica* Using Phylogenomics

Clarifying the taxonomic status of *A. cantabrica* has both scientific and conservation implications, as distinguishing it from closely related taxa is crucial for understanding its threatened status and prioritizing conservation measures (Godfray et al. 2004; Ottewell et al. 2016). Previous studies using morphological traits (Kress 1997), cytological counts (Kress 1984, 2022), plastid and nuclear markers (Schneeweiss et al. 2004), AFLPs (Dixon et al. 2008), plastome data (Smyčka et al. 2022), and ddRADSeq (Boucher et al. 2021) have proposed differing hypotheses regarding the placement of *A. cantabrica*, recovering varying relationships among *A. cantabrica*, *A. adfinis*, *A. halleri*, and *A. laggeri*. Here, we build on this foundational work by applying high‐resolution phylogenomic data from hundreds of nuclear loci and plastid sequences, which consistently resolve *A. cantabrica* as a monophyletic clade, confirming its phylogenetic distinctiveness.

The evolutionary origin of *A. cantabrica* has long been debated, with contrasting hypotheses based on morphology, cytology, and molecular evidence. Morphological comparisons previously aligned *A. cantabrica* most closely with *A. laggeri* (Kress 1997), while more recent treatments reaffirm its distinct morphotype and raise the possibility of local sympatry with *A. halleri* in the Peña Prieta region, an overlap that was considered unlikely (Kress 2022). Cytological data have added to the complexity: *A. cantabrica* was originally estimated to be an octoploid (2*n* ≈ 76), whereas *A. halleri* and *A. laggeri* are tetraploid (2*n* = 38; Kress 1984); however, misidentified specimens may have contributed to inconsistent chromosome counts (Kress 2022). In our study, sequence‐based ploidy inference using nQuire indicated that *A. cantabrica* is tetraploid, while both *A. halleri* and *A. laggeri* are diploid. Genome size estimates obtained by flow cytometry for the first time in *A. laggeri* and *A. cantabrica* confirm that the two species differ in ploidy. Moreover, comparison with data from the only ploidy screening to date in the genus, based on silica‐dried leaf material and involving different species (Dixon et al. 2009), corroborates the findings of the sequence‐based approach, likewise suggesting that *A. laggeri* is diploid and *A. cantabrica* tetraploid.

Molecular evidence has produced conflicting results. Earlier studies using *trn*L‐F and ITS sequences (Schneeweiss et al. 2004), AFLPs (Dixon et al. 2008), and ddRADseq (Boucher et al. 2021) recovered *A. cantabrica* and *A. adfinis* as sister taxa, forming a *cantabrica‐adfinis* clade that was itself sister to the /halleri clade comprising *A. halleri* and *A. laggeri*. However, these relationships were often weakly supported, and recent plastome‐based phylogenies have even questioned the sister relationship between the *cantabrica‐adfinis* clade and the /halleri clade (Smyčka et al. 2022). Our plastid data are congruent with previous studies in recovering *A. cantabrica* as sister to *A. adfinis*, supporting the *cantabrica‐adfinis* clade. However, our nuclear phylogenomic data from Angiosperms353 loci place *A. cantabrica* instead as sister to the /halleri clade, contradicting earlier nuclear and plastid studies and revealing a strong signal of cytonuclear discordance. This incongruence, common in plants, is often interpreted as the result of reticulate evolution, including hybridization, polyploidy, or incomplete lineage sorting (e.g., Rieseberg and Soltis 1991; Galbany‐Casals et al. 2014; Viruel et al. 2018; Favre et al. 2022; Liu et al. 2023).

Our data indicate that *A. cantabrica* is a tetraploid and likely has a complex evolutionary history. While Dixon et al. (2008) proposed an autopolyploid origin closely related to *A. adfinis*, our analysis suggests a different scenario, although we could not conclusively identify the exact polyploidy type. Although only a limited number of genes were flagged as paralogous in our dataset, which included *A. cantabrica* samples, this does not constitute definitive support for an autopolyploid origin. This low number of putative paralogues may instead reflect the close genetic relationships between the parental taxa, if *A. cantabrica* is indeed an allopolyploid, as homeologous sequences derived from phylogenetically related diploid ancestors may be difficult to distinguish using standard pipelines. For example, *A. adfinis* subsp. *brigantiaca* is suspected of being a recent tetraploid hybrid (Boucher et al. 2016), yet it only exhibited six paralogous genes in HybPiper analyses. Possible scenarios for the origin of *A. cantabrica* include an ancient homoploid hybridization event involving the ancestors of *A. cantabrica* and the /halleri clade, which may have been followed by whole genome duplication (i.e., allopolyploidization). Alternatively, hybridization could have occurred after polyploidization. Although our data cannot definitively resolve the sequence of these events, the observed phylogenetic conflict suggests that reticulate evolution involving hybridization followed by whole genome duplication likely played a role in the species' evolutionary history. In this context, the negative *F*_IS_ values observed across all *A. cantabrica* populations may be a consequence of allopolyploidy at the genomic level. Allopolyploid species often maintain fixed heterozygous loci inherited from divergent progenitor genomes, which can result in an apparent excess of heterozygotes and thus negative *F*_IS_ values. In any case, given its phylogenetic, morphological, and karyological uniqueness, *A. cantabrica* should be considered a valid species with an evolutionary trajectory shaped by rapid speciation, introgression, and possibly hybridization in alpine environments (Hibbins et al. 2020; Smyčka et al. 2022).

### 4.2 Conservation Status and Strategies for *Androsace cantabrica*

Our results support classifying *A. cantabrica* as Vulnerable (VU) based on the IUCN Red List framework, accounting for its restricted distribution, ongoing declines in area of occupancy, and quality of habitat. Our research indicates that the EOO is below 20,000 km^2^, with an AOO under 2000 km^2^, thus meeting the spatial thresholds for Vulnerable status. This reasoning is similar to that used to categorize *A. hemisphaerica* Ludlow as Endangered, given its very limited distribution range (i.e., EOO of 1008 km^2^; Bhutan Endemic Flowering Plants Workshop 2017). Furthermore, *A. cantabrica* populations are restricted to fewer than ten isolated locations, each experiencing habitat encroachment from shrub expansion and ongoing degradation due to human activities. One notable limitation of our IUCN assessment is the potential underestimation of population size due to the species' association with dense shrub margins, making it challenging to locate individuals challenging. The ongoing shrub expansion reduces the visibility of *A. cantabrica* and exacerbates competition for light and space, threatening population stability across its range. Additionally, while the current distribution data meets the IUCN's Vulnerable criteria, further expansion of shrub habitats (i.e., a lack of fire control to promote pastures) could eventually lead to an Endangered status. The potential decline in population size and the effects of human disturbances, such as ski resort maintenance, hiking trail use, or trampling, should be investigated more carefully. Such disturbances, combined with the environmental pressures from global warming and changes in land use in alpine zones, may increase the survival risk of the species. Open meadow habitats appear essential for *A. cantabrica* survival, and shrub encroachment and a decrease in herbivory pressure may negatively affect it throughout its entire distribution area. There is evidence of this, particularly in TM. These disturbances have a significant impact on the species' distribution and resilience, as reflected in the observed population declines.

The population genetic analysis divides *A. cantabrica* populations into two genetic conservation units: Group W, comprising western populations with lower genetic diversity, and Group E, which includes eastern populations showing relatively higher genetic diversity (*H*_E_ values as high as 0.2435). While the genetic data show a clear geographic structure between eastern and western populations, overall genetic differentiation among populations is low, and there is no evidence of inbreeding (as indicated by consistently negative *F*_IS_ values). Genetic diversity (*H*_E_) is moderate across populations, with slightly lower values observed in Group W. This may reflect the effect of smaller population sizes and greater habitat fragmentation in the western range, which could increase vulnerability to future genetic erosion. The negative *F*_IS_ values and the pattern of observed heterozygosity exceeding expected values are consistent with predominantly outcrossing reproduction. This is further supported by field observations of showy flowers and a diverse community of floral visitors. In addition, *A. cantabrica* is a tetraploid species, and polyploidy can contribute to high heterozygosity and an apparent excess of heterozygotes due to fixed differences between parental genomes.

For Group W, in situ conservation measures should focus on maintaining and restoring open alpine habitats suitable for *A. cantabrica*. In parallel, reinforcement of local populations through the planting of seedlings from locally collected seeds germinated in vitro (i.e., within‐population reinforcements) should be considered (Tejero et al. 2022). For the EP population, where both census size and genetic diversity are very low, such reinforcement strategies may be particularly valuable. Although translocation from genetically diverse populations could enhance genetic diversity in EP, this should be approached with caution, as it may disrupt local genetic structure even within Group W (Figure 3). Nevertheless, translocation could be explored as a last‐resort measure if demographic decline continues and should be preceded by careful experimental trials and genetic monitoring.

The Peña Prieta population is estimated to comprise approximately 1600 individuals, fragmented into smaller subpopulations, such as the LL population near Peña Prieta, which consists of approximately 100 individuals. Future work should involve gathering complementary genetic data from HC and Peña Prieta localities to identify potential donor populations with higher genetic diversity within the same genetic group and conducting translocations to strengthen the population size and genetic diversity in the EP population. Although the TM population exhibits the highest *H*_E_, translocating individuals from TM (i.e., Group E) to Group W is not advisable due to the potential risk of outbreeding depression (Lynch 1991). Avoiding translocations between genetically differentiated populations is crucial, as experiments investigating the risk of outbreeding depression are necessary to prevent adverse breeding effects (Liu and Zhao 1999). After any translocation efforts, if necessary, establishing a monitoring program to track fruiting rates, seed setting, and seedling survival will be essential for assessing population health and adaptability (Liu and Zhao 1999). Despite the relatively high *H*_E_ of the TM population, it is still lower than that of other non‐threatened taxa (e.g., *A. halleri* subsp. *nuria*), emphasizing the importance of mitigating anthropogenic disturbances in the TM area to preserve its genetic diversity. As an overall recommendation, conservation practices should focus on the genetic group W by reducing threats where appropriate and feasible. For example, this could involve reducing shrub competition to improve habitat suitability or collaborating with ski resorts near Peña Prieta to develop conservation and sustainable practices that mitigate human impact on surrounding habitats. Conversely, for Group E, conservation efforts should emphasize mitigating ecological threats by managing shrub encroachment to maintain habitat openness essential for *A. cantabrica* 's survival. Shrub encroachment poses a significant threat to the TM area, with impacts particularly severe at higher elevations where open habitats are more vulnerable to invasive shrub growth. This underscores the need for targeted interventions, such as controlled burning or grazing, where shrubs colonize high‐altitude habitats and limit suitable growing spaces for *A. cantabrica*. Additionally, we recommend long‐term monitoring of all known populations alongside efforts to locate and characterize additional populations.

*Ex situ* conservation approaches are efficient for the long‐term conservation of threatened species (e.g., Schoen and Brown 2001; Wambugu et al. 2023). In 2021, more than 2000 seeds were collected as part of the PRIOCONEX project (https://sites.google.com/aranzadi.eus/prioconex) to be stored at the Seed Bank in Gipuzkoa, Spain (Accession number 52/2020). Seeds from more than 50 mother plants were collected from the TM area to preserve most of its genetic diversity, and several morphometric measurements and germination protocols were conducted (Tejero et al. 2022). *Ex situ* conservation of seeds from the western group is also recommended; however, due to the scarcity of the species in the area, this task may be time‐ and resource‐intensive.

Micro‐reserves may be highly efficient for conserving cryptic populations of threatened plant species with minimal impact on local land use (Laguna et al. 2000; Médail et al. 2021). We recommend creating a micro‐reserve in a specific locality in the TM area (ETRS89 UN 86101 65592; 2058 MASL), which hosts the most conspicuous and dense known population and has the highest heterozygosity values. These actions align with the objectives of PRIOCONEX (Tejero et al. 2022), which focuses on *ex situ* conservation in response to climate impacts on alpine habitats (Yuste et al. 2021). However, its germination is strongly dependent on cold stratification. While current winter conditions in the studied localities may still be cold and dark enough to allow for dormancy breaking in most years, climate change is leading to shorter and warmer winters, reduced snowpack duration, and increased temperature variability. These shifts are expected to reduce the frequency and reliability of the cold, dark conditions required for natural germination (Körner 1999; Mondoni et al. 2012). This is particularly concerning for alpine plants in general (Fernández‐Pascual et al. 2021) and *A. cantabrica* specifically, whose populations are already located near the upper altitudinal limits of their respective mountain ranges. As a result, further upslope migration in response to warming is not possible, and reduced snow cover may impair seed dormancy release and germination timing, potentially limiting recruitment and threatening long‐term population viability. Additional research on the impact of environmental variability on germination and seedling establishment will be essential to guide conservation strategies under ongoing climate change.

Our results exemplify the potential of using target capture sequencing with the universal bait panel Angiosperms353 for population genetic studies (Slimp et al. 2021), offering high data quality and valuable cost efficiency for population‐level analyses. Angiosperms353 target capture has broad applications for conservation genetics, effectively capturing intraspecific variation within populations and supporting conservation genomics for rare or threatened taxa. The ability to integrate herbarium samples makes it particularly suited for conservation genetics (Slimp et al. 2021). Although Phang et al. (2023) found that population structure analysis using Angiosperms353 yielded limited resolution within species, our results demonstrate a higher resolution, likely because we did not combine multiple species when calling SNPs.

## 5 Conclusions

Our study confirms the taxonomic and phylogenetic distinctiveness of *A. cantabrica* and emphasizes the utility of Angiosperms353 target capture data in resolving species‐level conflicts within complex plant groups. We demonstrated that *A. cantabrica* is a distinct species that requires conservation action, contrary to previous hypotheses suggesting a close affiliation with *A. adfinis* subspecies. The contrasting results between nuclear and plastid phylogenies highlight the complex evolutionary history of *A. cantabrica* and related taxa, underlining the need for integrated molecular approaches to untangle rapid radiations and reticulate evolution. Future research should further investigate the polyploid origin of *A. cantabrica* and monitor its genetic structure and diversity in response to ongoing climate change. Long‐term conservation planning, including habitat management, controlled translocations, and *ex situ* conservation, will be vital to prevent genetic erosion and habitat loss for this Vulnerable alpine species.

The Angiosperms353 target capture approach proved effective for conservation genetics at the population level, even in polyploid species such as *A. cantabrica*. Moreover, we advocate for the adoption of Angiosperms353 in similar conservation genetics studies, given its cost‐effectiveness, sample efficiency, and the potential to incorporate herbarium samples while enabling comparative studies between species based on population genetic metrics calculated using the same set of molecular markers.

## Author Contributions

**Ke (Jungle) Liang:** formal analysis (lead), writing – original draft (equal). **Amelia Shepherd‐Clowes:** investigation (equal). **Agustí Agut:** investigation (equal). **Oriane Hidalgo:** investigation (equal), writing – review and editing (equal). **Pablo Tejero Ibarra:** conceptualization (lead), funding acquisition (equal), investigation (equal), writing – review and editing (equal). **Juan Viruel:** formal analysis (supporting), funding acquisition (equal), investigation (equal), supervision (lead), writing – original draft (equal), writing – review and editing (equal).

## Conflicts of Interest

The authors declare no conflicts of interest.

## Supporting information


**Figure S1:** ece371901‐sup‐0001‐FiguresS1‐S2.pdf.

**Table S1:** ece371901‐sup‐0002‐TablesS1‐S3.docx.

## Data Availability

Data uploaded to ENA repository PRJEB90944. All the required data are uploaded as [Link], [Link].
