# Supplementary material for: Conservation Implications for the Iberian Narrow Endemic Androsace cantabrica (Primulaceae) Using Population Genomics With Target Capture Sequence Data
Source: Ecol Evol. 2025 Aug 8;15(8):e71901. doi: 10.1002/ece3.71901 (PMC12334361; doi:10.1002/ece3.71901)
Supplement: Supplementary file 1 — Figure S1: ece371901‐sup‐0001‐FiguresS1‐S2.pdf. [file ECE3-15-e71901-s002.pdf]

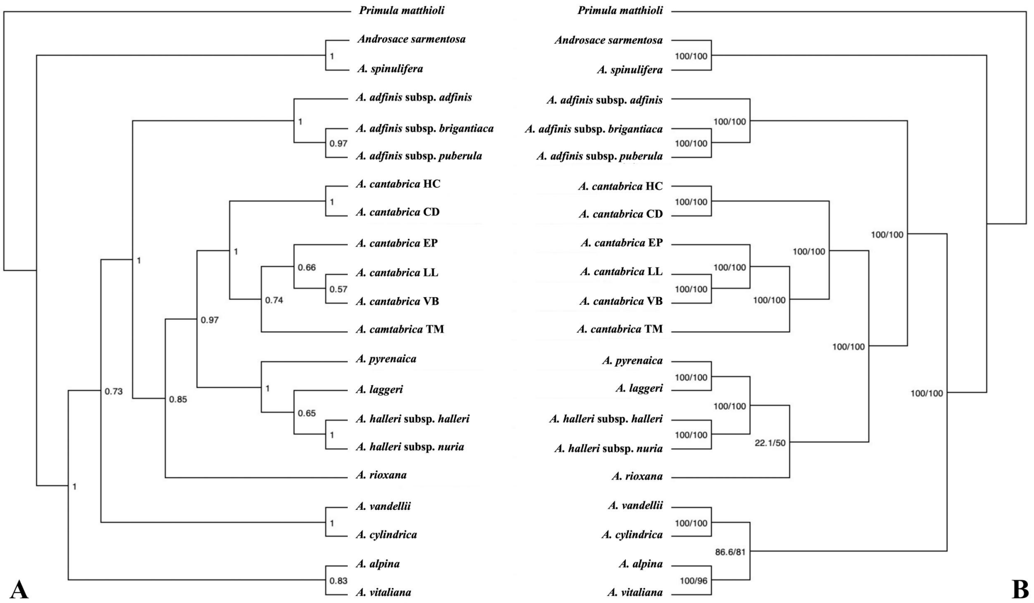

Figure 1. The comparison of the topology of Angiosperms353 loci astral tree (A) and IQ partition tree (B). (A) The astral tree was generated based on the coalescent method, with the values of the nodes representing local posterior probabilities (LPP). (B) The IQ partition tree was generated in IQ-Tree with the partition model solution based on the concatenated matrix using the ML method, with the values of the nodes representing support for 1000 SH-like approximate likelihood ratio test (left) and ultrafast bootstrap (right) replicates.

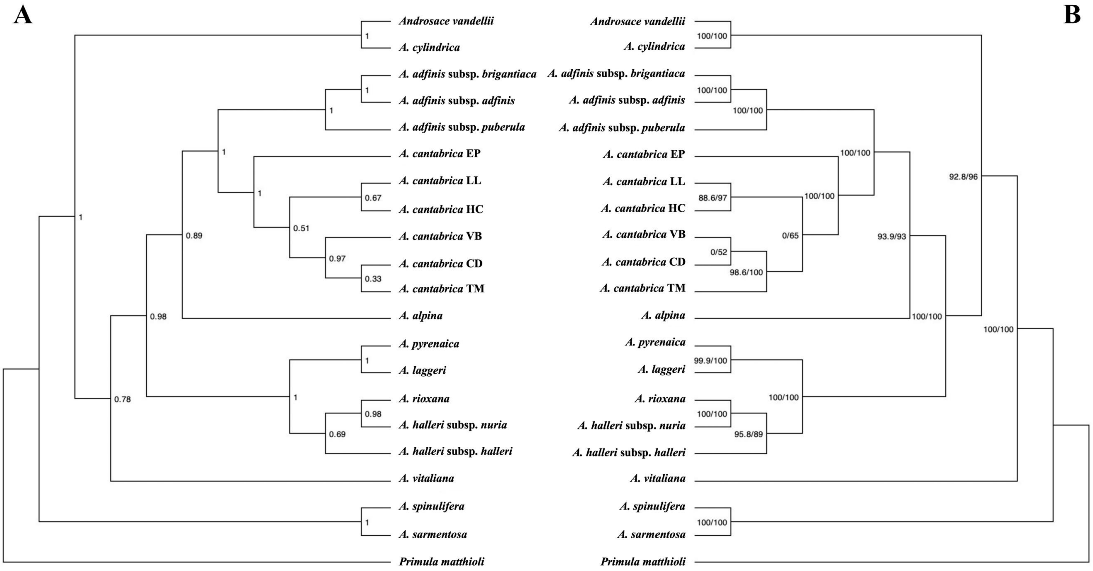

Figure 2. The comparison of the topology of 125 plastid fragments astral tree (A) and IQ partition tree (B). (A) The astral tree was generated based on the coalescent method, with the values of the nodes representing local posterior probabilities (LPP). (B) The IQ partition tree was generated in IQ-Tree with the partition model solution based on the concatenated matrix using the ML method, with the values of the nodes representing support for 1000 SH-like approximate likelihood ratio test (left) and ultrafast bootstrap (right) replicates.
