# Supplementary material for: Conservation Implications for the Iberian Narrow Endemic Androsace cantabrica (Primulaceae) Using Population Genomics With Target Capture Sequence Data
Source: Ecol Evol. 2025 Aug 8;15(8):e71901. doi: 10.1002/ece3.71901 (PMC12334361; doi:10.1002/ece3.71901)
Supplement: Supplementary file 2 — Table S1: ece371901‐sup‐0002‐TablesS1‐S3.docx. [file ECE3-15-e71901-s001.docx]

**Table S1.** Statistics of sequencing data, target capture and sequence length recovery generated by Angiosperms353 sequencing using the mega353 target file. The last four raw sequencing data were obtained through online downloads. ^1^Mapped Reads represents reads on the Angiosperms353 loci target. "Genes at 50%" represents the number of genes with sequence recovery lengths of 50% or more of the average sequence length of the target.

| **Sequencing Code / SRA** | **Species** | **Raw Reads** | **Trimmed**  **Reads** | **Mapped Reads^1^** | **% on Tagert** | **Genes at 50%** | **Seq Length** | **% Seq Length Recovery** |
| --- | --- | --- | --- | --- | --- | --- | --- | --- |
| 21B47 | *A. cantabrica* | 2763208 | 2754552 | 819582 | 29.75% | 306 | 236424 | 82.20% |
| 21B48 | *A. cantabrica* | 4041414 | 4039848 | 608722 | 15.07% | 300 | 226194 | 78.64% |
| 21B49 | *A. cantabrica* | 3986046 | 3978166 | 1590660 | 39.98% | 316 | 234810 | 81.63% |
| 21B50 | *A. cantabrica* | 2861552 | 2861126 | 771077 | 26.95% | 308 | 232821 | 80.94% |
| 21B51 | *A. cantabrica* | 1931212 | 1924556 | 478870 | 24.88% | 300 | 223806 | 77.81% |
| 21B52 | *A. cantabrica* | 3353306 | 3335236 | 834894 | 25.03% | 311 | 231228 | 80.39% |
| 21B53 | *A. cantabrica* | 3101410 | 3098914 | 754920 | 24.36% | 310 | 244515 | 85.01% |
| 21B54 | *A. cantabrica* | 2054584 | 2052840 | 418680 | 20.40% | 292 | 221814 | 77.12% |
| 21B55 | *A. cantabrica* | 2595410 | 2593916 | 728147 | 28.07% | 311 | 232509 | 80.83% |
| 21B56 | *A. cantabrica* | 4346382 | 4345666 | 995983 | 22.92% | 318 | 240357 | 83.56% |
| 21B57 | *A. cantabrica* | 5076276 | 5072778 | 1398697 | 27.57% | 318 | 241968 | 84.12% |
| 21B58 | *A. cantabrica* | 7209994 | 7191142 | 2437026 | 33.89% | 329 | 247761 | 86.14% |
| 21B59 | *A. cantabrica* | 4343438 | 4335564 | 1236768 | 28.53% | 317 | 243261 | 84.57% |
| 21B60 | *A. cantabrica* | 5162880 | 5141346 | 1581437 | 30.76% | 322 | 243030 | 84.49% |
| 21B61 | *A. cantabrica* | 2612 | 2606 | 704 | 27.01% | 0 | 0 | 0.00% |
| 21B62 | *A. cantabrica* | 3364936 | 3363508 | 932321 | 27.72% | 313 | 239547 | 83.28% |
| 21B63 | *A. cantabrica* | 4366022 | 4349834 | 1298362 | 29.85% | 319 | 239967 | 83.43% |
| 21B64 | *A. cantabrica* | 4320806 | 4320138 | 948078 | 21.95% | 311 | 237822 | 82.68% |
| 21B65 | *A. cantabrica* | 2763198 | 2761310 | 769072 | 27.85% | 313 | 233916 | 81.32% |
| 21B66 | *A. cantabrica* | 5235514 | 5222278 | 989478 | 18.95% | 314 | 234876 | 81.66% |
| 21B67 | *A. cantabrica* | 4989658 | 4963382 | 1256776 | 25.32% | 315 | 235635 | 81.92% |
| 21B68 | *A. cantabrica* | 6723012 | 6665700 | 1521919 | 22.83% | 318 | 230961 | 80.30% |
| 21B69 | *A. cantabrica* | 4631680 | 4627894 | 965922 | 20.87% | 306 | 237024 | 82.40% |
| 21B86 | *A. cantabrica* | 3076054 | 3074834 | 1168381 | 38.00% | 317 | 239961 | 83.43% |
| 21B87 | *A. cantabrica* | 3326840 | 3322442 | 1003844 | 30.21% | 318 | 239919 | 83.41% |
| 21B88 | *A. cantabrica* | 4167690 | 4167456 | 969129 | 23.25% | 315 | 238503 | 82.92% |
| 21B89 | *A. cantabrica* | 3670400 | 3665004 | 1117340 | 30.49% | 315 | 240111 | 83.48% |
| 21B90 | *A. cantabrica* | 3087416 | 3086300 | 799238 | 25.90% | 318 | 248874 | 86.52% |
| Z1 | *A. cantabrica* | 14042070 | 14041134 | 7362081 | 52.43% | 323 | 243735 | 84.74% |
| Z2 | *A. cantabrica* | 15335242 | 15334188 | 8763126 | 57.15% | 330 | 247020 | 85.88% |
| Z4 | *A. cantabrica* | 73658 | 73646 | 35177 | 47.76% | 58 | 73500 | 25.55% |
| Z5 | *A. cantabrica* | 19161442 | 19157526 | 9356454 | 48.84% | 325 | 246756 | 85.79% |
| Z6 | *A. cantabrica* | 20493920 | 20491392 | 1032661 | 50.39% | 328 | 244086 | 84.86% |
| Z7 | *A. cantabrica* | 13644860 | 13643024 | 7568027 | 55.47% | 324 | 243576 | 84.68% |
| Z8 | *A. cantabrica* | 15611912 | 15610714 | 8915060 | 57.11% | 323 | 244755 | 85.09% |
| 21B70 | *A. laggeri* | 3255644 | 3246132 | 482918 | 14.88% | 294 | 222324 | 77.29% |
| 21B79 | *A. halleri* subsp. *nuria* | 2162607 | 2155763 | 828977 | 38.45% | 304 | 228624 | 79.48% |
| 21B80 | *A. halleri* subsp. *nuria* | 2215219 | 2215158 | 568470 | 25.66% | 308 | 234885 | 81.66% |
| 21B81 | *A. halleri* subsp. *nuria* | 2275813 | 2275452 | 597725 | 26.27% | 305 | 232587 | 80.86% |
| 21B82 | *A. halleri* subsp. *nuria* | 2249069 | 2248515 | 949938 | 42.25% | 316 | 242340 | 84.25% |
| 21B83 | *A. halleri* subsp. *nuria* | 1857335 | 1856668 | 779653 | 41.99% | 312 | 236103 | 82.08% |
| 21B84 | *A. halleri* subsp. *nuria* | 4274102 | 4270144 | 1030756 | 24.14% | 317 | 243261 | 84.57% |
| 21B85 | *A. halleri* subsp. *halleri* | 2455782 | 2454096 | 821208 | 33.46% | 318 | 239226 | 83.17% |
| 21B44 | *A. vandellii* | 7169544 | 7162908 | 1286541 | 17.6% | 322 | 242094 | 84.17% |
| 21B45 | *A. pyrenaica* | 3183770 | 3181660 | 1059074 | 33.29% | 317 | 234744 | 81.61% |
| 21B46 | *A. cylindirca* subsp. *hirtella* | 3290838 | 3289172 | 943726 | 28.69% | 317 | 238947 | 83.07% |
| 21E24 | *A. rioxana* | 4059594 | 4042674 | 726445 | 17.97% | 285 | 206991 | 71.96% |
| 23H53 | *A. adfinis* subsp. *adfinis* | 8599046 | 8592758 | 5742960 | 66.83% | 316 | 235146 | 81.75% |
| 23H55 | *A. adfinis* subsp. *puberula* | 4468184 | 4466064 | 2222771 | 49.77% | 249 | 181701 | 63.17% |
| 23H57 | *A. adfinis* subsp. *brigantiaca* | 5346300 | 5344540 | 3428863 | 64.16% | 291 | 208992 | 72.66% |
| 23H58 | *A. alpina* | 6233538 | 6231496 | 2422193 | 38.87% | 294 | 216540 | 75.28% |
| ERR7620526 | *A. sarmentosa* | 2668748 | 2668510 | 519444 | 19.47% | 210 | 165369 | 57.49% |
| ERR7620605 | *A. vitaliana* | 1438832 | 1438554 | 625155 | 43.46% | 245 | 183372 | 63.75% |
| SRR19354411 | *A. spinulifera* | 60942 | 60364 | 18137 | 30.05% | 20 | 35001 | 12.17% |
| ERR7620530 | *Primula matthioli* | 2629710 | 2629526 | 937636 | 35.66% | 215 | 156498 | 54.41% |

**Table S2.** Statistics of sequencing data and sequence length recovery generated by Angiosperms353 off-target data or genome skimming sequencing data using the 125 plastid fragments target file. The last eight raw sequencing data were obtained through online downloads.

| **Sequencing Code / SRA** | **Species** | **Raw Reads** | **Trimmed Reads** | **Mapped Reads** | **Seq**  **Length** | **% Seq length Recovery** |
| --- | --- | --- | --- | --- | --- | --- |
| 21B56 | *A. cantabrica* | 4346382 | 4345666 | 93811 | 150318 | 96.19% |
| 21B58 | *A. cantabrica* | 7209994 | 7191142 | 238450 | 154149 | 98.64% |
| 21B63 | *A. cantabrica* | 4366022 | 4349834 | 201157 | 153975 | 98.53% |
| 21B88 | *A. cantabrica* | 4167690 | 4167456 | 31348 | 94845 | 60.69% |
| Z6 | *A. cantabrica* | 20493920 | 20491392 | 346079 | 152127 | 97.35% |
| Z8 | *A. cantabrica* | 15611912 | 15610714 | 147592 | 140043 | 89.61% |
| 21B70 | *A. laggeri* | 3255644 | 3246132 | 195940 | 154092 | 98.60% |
| 21B84 | *A. halleri* subsp. *nuria* | 4274102 | 4270144 | 51290 | 113214 | 72.45% |
| 21B85 | *A. halleri* subsp. *halleri* | 2455782 | 2454096 | 73496 | 141642 | 90.64% |
| 21B44 | *A. vandellii* | 7169544 | 7162908 | 50493 | 153741 | 98.38% |
| 21B45 | *A. pyrenaica* | 3183770 | 3181660 | 108817 | 154086 | 98.60% |
| 21B46 | *A. cylindirca* subsp. *hirtella* | 3290838 | 3289172 | 73605 | 153996 | 98.54% |
| 21E24 | *A. rioxana* | 4059594 | 4042674 | 176049 | 153459 | 98.20% |
| 23H53 | *A. adfinis* subsp. *adfinis* | 4299523 | 4296379 | 135450 | 66492 | 42.55% |
| 23H55 | *A. adfinis* subsp. *puberula* | 2234092 | 2233032 | 43698 | 15510 | 9.93% |
| 23H57 | *A. adfinis* subsp. *brigantiaca* | 2673150 | 2672270 | 81739 | 54051 | 34.59% |
| 23H58 | *A. alpina* | 6233538 | 6231496 | 73793 | 43371 | 27.75% |
| ERR7620526 | *A. sarmentosa* subsp. *primuloides* | 2668748 | 2668510 | 24203 | 79359 | 50.78% |
| ERR7620605 | *A. vitaliana* | 1438832 | 1438554 | 15411 | 40239 | 25.75% |
| ERR9124249 | *A. adfinis* subsp. *adfinis* | 6494828 | 6494812 | 421949 | 154188 | 98.67% |
| ERR9124250 | *A. adfinis* subsp. *brigantiaca* | 4055448 | 4055430 | 279139 | 154206 | 98.68% |
| ERR9124251 | *A. adfinis* subsp. *puberula* | 10420686 | 10420582 | 875600 | 154200 | 98.67% |
| ERR9124252 | *A. alpina* | 7116370 | 7116280 | 455556 | 154203 | 98.68% |
| SRR19401086 | *A. spinulifera* | 3032236 | 2778296 | 42009 | 138981 | 88.94% |
| ERR7620530 | *Primula matthioli* | 2629710 | 2629526 | 21413 | 52044 | 33.30% |

**Table S3.** Results of ploidy estimation for *Androsace* individuals belonging to different taxa. "Genes with paralog warning" represents the number of genes given paralog warnings for each sample by Hybpiper; "log*L* free" represents the value of the free model maximized log-likelihood in nQuire; "M" represents the three ploidy models in nQuire: (2x) diploid, (3x) triploid, and (4x) tetraploid; "log*L*" represents the value of the fixed model under three models; "*Δ*log*L*" represents the value of delta log-likelihood under three models; those marked in green are the best ploidy estimation for each sample inferred from the lowest "*Δ*log*L*" value under three models.

| **Sequencing**  **Code** | **Species** | **Genes with paralog warnings** | **log*L* free** | **M** | **log*L*** | ***Δ*log*L*** |  |
| --- | --- | --- | --- | --- | --- | --- | --- |
| 21B47 | *A. cantabrica* | 30 | 2402.429918 | 2x | 581.176227 | 1821.253690 | |
|  |  |  |  | 3x | 1017.904675 | 1384.525243 | |
|  |  |  |  | **4x** | **2341.346781** | **61.083137** | |
| 21B48 | *A. cantabrica* | 22 | 3928.505323 | 2x | 1969.738332 | 1958.766991 | |
|  |  |  |  | 3x | 2734.280874 | 1194.224449 | |
|  |  |  |  | **4x** | **3357.608318** | **570.897005** | |
| 21B49 | *A. cantabrica* | 26 | 1568.623995 | 2x | 415.292478 | 1153.331517 | |
|  |  |  |  | 3x | 899.283970 | 669.340025 | |
|  |  |  |  | **4x** | **1465.247828** | **103.376167** | |
| 21B50 | *A. cantabrica* | 27 | 3594.443608 | 2x | 1938.129002 | 1656.314606 | |
|  |  |  |  | 3x | 2543.496541 | 1050.947067 | |
|  |  |  |  | **4x** | **2979.534307** | **614.909301** | |
| 21B51 | *A. cantabrica* | 28 | 2113.969380 | 2x | 337.879149 | 1776.090231 | |
|  |  |  |  | 3x | 972.076073 | 1141.893306 | |
|  |  |  |  | **4x** | **2079.542632** | **34.426747** | |
| 21B52 | *A. cantabrica* | 26 | 2541.553351 | 2x | 511.486564 | 2030.066788 | |
|  |  |  |  | 3x | 1099.066959 | 1442.486393 | |
|  |  |  |  | **4x** | **2446.296612** | **95.256740** | |
| 21B53 | *A. cantabrica* | 38 | 4032.824979 | 2x | 2258.514990 | 1774.309989 | |
|  |  |  |  | 3x | 2850.189514 | 1182.635465 | |
|  |  |  |  | **4x** | **3288.782214** | **744.042765** | |
| 21B54 | *A. cantabrica* | 29 | 1097.336987 | 2x | 284.014632 | 813.322355 | |
|  |  |  |  | 3x | 689.009128 | 408.327859 | |
|  |  |  |  | **4x** | **1016.051347** | **81.285640** | |
| 21B55 | *A. cantabrica* | 28 | 2438.297040 | 2x | 761.286862 | 1677.010179 | |
|  |  |  |  | 3x | 1225.824579 | 1212.472461 | |
|  |  |  |  | **4x** | **2338.930874** | **99.366167** | |
| 21B56 | *A. cantabrica* | 33 | 2687.333092 | 2x | 606.001997 | 2081.331095 | |
|  |  |  |  | 3x | 1106.253163 | 1581.079930 | |
|  |  |  |  | **4x** | **2528.721765** | **158.611328** | |
| 21B57 | *A. cantabrica* | 29 | 2172.562241 | 2x | 658.528238 | 1514.034003 | |
|  |  |  |  | 3x | 1159.676734 | 1012.885507 | |
|  |  |  |  | **4x** | **1981.530953** | **191.031288** | |
| 21B58 | *A. cantabrica* | 35 | 2631.091115 | 2x | 666.409115 | 1964.682000 | |
|  |  |  |  | 3x | 1132.267336 | 1498.823779 | |
|  |  |  |  | **4x** | **2536.794196** | **94.296919** | |
| 21B59 | *A. cantabrica* | 32 | 3551.156531 | 2x | 1614.931131 | 1936.225400 | |
|  |  |  |  | 3x | 2442.811970 | 1108.344562 | |
|  |  |  |  | **4x** | **3096.232391** | **454.924141** | |
| 21B60 | *A. cantabrica* | 33 | 2584.911951 | 2x | 589.558145 | 1995.353806 | |
|  |  |  |  | 3x | 1033.734296 | 1551.177655 | |
|  |  |  |  | **4x** | **2535.398818** | **49.513133** | |
| 21B62 | *A. cantabrica* | 35 | 3646.790454 | 2x | 1043.863827 | 2602.926627 | |
|  |  |  |  | 3x | 1877.113247 | 1769.677206 | |
|  |  |  |  | **4x** | **3385.734045** | **261.056409** | |
| 21B63 | *A. cantabrica* | 28 | 2403.933081 | 2x | 537.197456 | 1866.735625 | |
|  |  |  |  | 3x | 1237.295084 | 1166.637997 | |
|  |  |  |  | **4x** | **2331.809567** | **72.123514** | |
| 21B64 | *A. cantabrica* | 36 | 2973.859283 | 2x | 656.665345 | 2317.193938 | |
|  |  |  |  | 3x | 1175.560473 | 1798.298809 | |
|  |  |  |  | **4x** | **2885.383293** | **88.475990** | |
| 21B65 | *A. cantabrica* | 27 | 2649.724394 | 2x | 697.622647 | 1952.101747 | |
|  |  |  |  | 3x | 1213.218338 | 1436.506056 | |
|  |  |  |  | **4x** | **2589.115559** | **60.608834** | |
| 21B66 | *A. cantabrica* | 23 | 1525.609073 | 2x | 505.978051 | 1019.631022 | |
|  |  |  |  | 3x | 810.325797 | 715.283276 | |
|  |  |  |  | **4x** | **1401.724003** | **123.885070** | |
| 21B67 | *A. cantabrica* | 28 | 4019.657859 | 2x | 1780.923835 | 2238.734024 | |
|  |  |  |  | 3x | 2455.169126 | 1564.488733 | |
|  |  |  |  | **4x** | **3611.483230** | **408.174629** | |
| 21B68 | *A. cantabrica* | 22 | 1512.942390 | 2x | 468.263759 | 1044.678631 | |
|  |  |  |  | 3x | 789.289344 | 723.653046 | |
|  |  |  |  | **4x** | **1454.393419** | **58.548970** | |
| 21B69 | *A. cantabrica* | 27 | 2321.465874 | 2x | 460.155493 | 1861.310381 | |
|  |  |  |  | 3x | 980.235351 | 1341.230523 | |
|  |  |  |  | **4x** | **2285.426698** | **36.039176** | |
| 21B86 | *A. cantabrica* | 31 | 2801.781716 | 2x | 936.376941 | 1865.404775 | |
|  |  |  |  | 3x | 1208.876091 | 1592.905625 | |
|  |  |  |  | **4x** | **2688.004056** | **113.777660** | |
| 21B87 | *A. cantabrica* | 34 | 2765.256786 | 2x | 860.737155 | 1904.519631 | |
|  |  |  |  | 3x | 1263.214563 | 1502.042224 | |
|  |  |  |  | **4x** | **2485.363579** | **279.893208** | |
| 21B88 | *A. cantabrica* | 34 | 2978.722841 | 2x | 678.001289 | 2300.721553 | |
|  |  |  |  | 3x | 1272.088600 | 1706.634242 | |
|  |  |  |  | **4x** | **2922.858519** | **55.864322** | |
| 21B89 | *A. cantabrica* | 35 | 2666.933231 | 2x | 686.818631 | 1980.114599 | |
|  |  |  |  | 3x | 1216.504205 | 1450.429025 | |
|  |  |  |  | **4x** | **2602.240378** | **64.692852** | |
| 21B90 | *A. cantabrica* | 62 | 2245.375283 | 2x | 502.494725 | 1742.880557 | |
|  |  |  |  | 3x | 1111.310893 | 1134.064389 | |
|  |  |  |  | **4x** | **2096.046643** | **149.328640** | |
| Z1 | *A. cantabrica* | 25 | 1792.417922 | 2x | 275.988474 | 1516.429448 | |
|  |  |  |  | 3x | 1047.024087 | 745.393835 | |
|  |  |  |  | **4x** | **1722.596774** | **69.821148** | |
| Z2 | *A. cantabrica* | 22 | 1717.306207 | 2x | 268.168789 | 1449.137418 | |
|  |  |  |  | 3x | 833.603129 | 883.703078 | |
|  |  |  |  | **4x** | **1678.797855** | **38.508352** | |
| Z4 | *A. cantabrica* | - | 5266.554700 | 2x | 200.487178 | 5066.067522 | |
|  |  |  |  | 3x | 457.421713 | 4809.132987 | |
|  |  |  |  | **4x** | **606.306826** | **4660.247873** | |
| Z5 | *A. cantabrica* | 17 | 1538.774817 | 2x | 179.567689 | 1359.207128 | |
|  |  |  |  | 3x | 706.487135 | 832.287681 | |
|  |  |  |  | **4x** | **1497.466536** | **41.308281** | |
| Z6 | *A. cantabrica* | 19 | 1897.837878 | 2x | 159.246701 | 1738.591177 | |
|  |  |  |  | 3x | 757.872072 | 1139.965806 | |
|  |  |  |  | **4x** | **1839.586049** | **58.251829** | |
| Z7 | *A. cantabrica* | 17 | 1512.367363 | 2x | 287.173940 | 1225.193423 | |
|  |  |  |  | 3x | 782.403227 | 729.964136 | |
|  |  |  |  | **4x** | **1485.796316** | **26.571047** | |
| Z8 | *A. cantabrica* | 21 | 1724.294155 | 2x | 372.203923 | 1352.090232 | |
|  |  |  |  | 3x | 1023.372736 | 700.921419 | |
|  |  |  |  | **4x** | **1623.796884** | **100.497271** | |
| 23H53 | *A. adfinis* subsp*. adfinis* | 20 | 3986.397947 | **2x** | **3870.398846** | **115.999101** | |
|  |  |  |  | 3x | 2038.621332 | 1947.776615 | |
|  |  |  |  | 4x | 2060.946544 | 1925.451403 | |
| 23H55 | *A. adfinis* subsp*. puberula* | 5 | 1567.860590 | **2x** | **1525.104956** | **42.755634** | |
|  |  |  |  | 3x | 922.017602 | 645.842988 | |
|  |  |  |  | 4x | 757.217412 | 810.643178 | |
| 23H57 | *A. adfinis* subsp*. brigantiaca* | 6 | 504.451322 | 2x | 213.821106 | 290.630216 | |
|  |  |  |  | 3x | 378.383637 | 126.067685 | |
|  |  |  |  | **4x** | **395.476523** | **108.974799** | |
| 21B70 | *A. laggeri* | 28 | 3359.542295 | **2x** | **3284.866197** | **74.676097** | |
|  |  |  |  | 3x | 1597.366075 | 1762.176220 | |
|  |  |  |  | 4x | 1779.391186 | 1580.151109 | |
| 21B84 | *A. halleri subsp. nuria* | 39 | 4393.671851 | **2x** | **4136.989241** | **256.682610** | |
|  |  |  |  | 3x | 1504.717538 | 2888.954313 | |
|  |  |  |  | 4x | 2637.658819 | 1756.013032 | |
| 21B85 | *A. halleri subsp. halleri* | 28 | 4393.150548 | **2x** | **4270.270710** | **122.879838** | |
|  |  |  |  | 3x | 2014.289798 | 2378.860750 | |
|  |  |  |  | 4x | 2362.511085 | 2030.639463 | |
| 21B45 | *A. pyrenaica* | 20 | 3141.613878 | **2x** | **3017.758540** | **123.855338** | |
|  |  |  |  | 3x | 1777.428594 | 1364.185284 | |
|  |  |  |  | 4x | 1655.253350 | 1486.360528 | |
| 21E24 | *A. rioxana* | 11 | 1230.610642 | 2x | 233.714441 | 996.896201 | |
|  |  |  |  | 3x | 657.721093 | 996.896201 | |
|  |  |  |  | **4x** | **1135.352580** | **95.258062** | |
